# The impact of host genetics on porcine gut microbiota composition excluding maternal and postnatal environmental influences

**DOI:** 10.1371/journal.pone.0315199

**Published:** 2024-12-09

**Authors:** Ana Heras-Molina, Jordi Estellé, Marta Vázquez-Gómez, Adrián López-García, José-Luis Pesantez-Pacheco, Susana Astiz, Consolación Garcia-Contreras, Rosa Escudero, Beatriz Isabel, Antonio Gonzalez-Bulnes, Cristina Óvilo

**Affiliations:** 1 Faculty of Veterinary Medicine, UCM, Ciudad Universitaria s/n, Madrid, Spain; 2 CSIC-INIA, Madrid, Spain; 3 Université Paris-Saclay, INRAE, AgroParisTech, GABI, Jouy-en-Josas, France; 4 Sorbonne université, INSERM, Nutrition et obésités: approaches systémiques, Nutriomics, Paris, France; 5 School of Veterinary Medicine and Zootechnics, Faculty of Agricultural Sciences, University of Cuenca, Cuenca, Ecuador; 6 Faculty of Veterinary Medicine, Universidad Cardenal Herrera-CEU, CEU Universities, Valencia, Spain; University of Life Sciences in Lublin, POLAND

## Abstract

The gut microbiota of the pig is being increasingly studied due to its implications for host homeostasis and the importance of the pig as a meat source and biomedical model of human diseases. However, most studies comparing the microbiome between different breeds do not consider the influence of maternal environment during the colonization of the microbiota. The aim of the present study was to compare the gut microbiota during postnatal growth between two pig genotypes (purebred Iberian *vs*. crossbreds Iberian x Large White pigs), gestated in a single maternal environment (pure Iberian mothers) inseminated with heterospermic semen. Postnatally, piglets were maintained in the same environmental conditions, and their microbiota was studied at 60 and 210 days old. Results showed that age had the greatest influence on alpha and beta diversity, and genotype also affected beta diversity at both ages. There were differences in the microbiome profile between genotypes at the ASV and genus levels when jointly analyzing the total number of samples, which may help to explain phenotypical differences. When each time-point was analyzed individually, there were more differences at 210 days-old than 60 days-old. Fecal short-chain fatty acids (SCFA) were also affected by age, but not by genotype. These results may be a basis for further research on host genotype interactions with the gut microbiota.

## Introduction

Gastrointestinal tract is colonized by a diverse microbiota population, which exerts various biological functions on the animal through a symbiotic relationship [1]. The importance of gut microbiota is widely recognized, often referred to as the ’lost organ’ of the host [2]. In pigs, microbiota has been increasingly studied due to the economic relevance of porcine production [3] since pork is one of the most consumed meats worldwide [4]. In addition, the pig’s gastrointestinal tract is closer to humans’ than other animal models, such as mice, being a better biomedical model for digestive and metabolic diseases [5, 6]. The gut microbiota is known to exert important functions in nutrient digestibility and absorption [7, 8], vitamin synthesis [9] and prevention of infections and inflammation by short-chain fatty acid (SCFA) production [10, 11].

There is also a wide variety of porcine breeds and genotypes that have evolved differently during the past decades. Commercial breeds, such as Large White, have been selected for meat production and, therefore, have high growth rates and feed efficiency levels, as well as improved reproductive parameters [12, 13]. On the other hand, traditional breeds, such as the Iberian pig, are rustic animals with high adiposity and low growth efficiency and fertility [14]. These differences between breeds allow us to obtain meat products with different properties and qualities [15, 16].

Host genome has been previously linked to microbiome composition in pigs [17, 18]. Concretely, in pigs *Akkermansia* abundance has been previously associated with polymorphisms in five chromosomic regions (SSC3, SSC6, SSC7, SSC9, and SSC15) while *Prevotella* was associated with the single nucleotide polymorphism (SNP) *rs326174858* [17]. Previous research also found an important effect of the pig breed on the richness and microbiome composition, which were related to productivity and meat characteristics [18, 19].

Various studies focus on the differences between the Iberian and lean pig breeds to understand the breed-microbiota relationships and their connexion to productivity [17, 18, 20]. However, in these studies, the piglets were gestated in their corresponding obese or lean mothers. Thus, the maternal influence cannot be completely left aside since it largely influences neonatal primocolonization, with long-lasting functional effects during the offspring’s life [21, 22].

The aim of the present study was to determine the differences of the microbiome composition between pig genotypes independently of confounding factors related to the maternal and postnatal environment. The Iberian breed was used to determine such relative roles of genetics and environment by comparing two genotypes (purebred Iberian and crossbreds Iberian x Large White), produced in one single maternal environment (pure Iberian mothers) through artificial insemination of Iberian sows with Iberian and Large White heterospermic semen and maintained in the same environmental conditions during postnatal development.

## Materials and methods

### Ethic statement

The experiment was assessed and approved by the INIA-CSIC Committee of Ethics in Animal Research (report CEEA 2013/036), and subsequently by the regional competent authority (report PROEX114/16), according to the Spanish Policy for Animal Protection (RD 53/2013) which meets the European Union Directive 2010/63/UE on the protection of research animals.

### Animals and experimental design

Phenotypic features, environmental conditions, nutrition and handling of sows, boars and piglets have been previously described [23]. In brief, 16 purebred multiparous (2–3 parity) Iberian sows were kept at the intensive farm Ibericos de Arauzo S.L. (Zorita de la Frontera, Salamanca, Spain). They were synchronized with altrenogest (Regumate, MSD, Boxmeer, The Netherlands). Afterwards, they were inseminated with heterospermic seminal doses obtained by mixing semen from two purebred Iberian (183 and 197 kg of body-weight) and two purebred Large White boars (324 and 352 kg of body-weight). After collection, the semen quality was evaluated (sperm concentration, morphology and motility) and the ejaculates were mixed at equal viable spermatozoa concentrations for Iberian and Large White fractions and aliquoted into 80 mL doses containing 6 × 10^9^ viable spermatozoa.

All sows and boars were previously genotyped by pyrosequencing using blood samples to confirm homozygosity for the LEPRc.1987C/T polymorphism. Allele LEPRc. 1987T is fixed in the Iberian breed, so it can be used for genotype confirmation with Iberians being TT and crossbreds being CT [24].

The sows were fed a standard grain-based diet (89.9% of dry matter, 13% of crude protein, 2.6% of fat and 2.2 Mcal/kg of metabolizable energy; S1 Table) to fulfill individual pregnancy and lactation requirements based on data from the National Research Council [25].

At birth, the total number of piglets (both alive and stillborn) was recorded for each sow, and living piglets were sampled for ascertaining homo- or heterozygosity for the *LEPR* gene by pyrosequencing using blood samples. Pure Iberian pigs were LEPRc.1987T/T (IBxIB), whereas Iberian*Large White crossbreds were LEPRc.1987C/T (IBxLW). All living piglets were ear-tagged for identification. During the first week of life, male piglets were castrated following the RD 1135/2002, under standard handling practices.

Piglets remained with the sows in individual pens until weaning at the age of 21 days-old, when they were moved to collective pens mixing genotypes and litters. The room temperature was 26°C and piglets were fed with a standard commercial diet (89.5% of dry matter, 15% of crude protein, 4% of fat and 2.4 Mcal/kg of metabolizable energy; S2 Table) adjusted to fulfill growing requirements. At 60 days-old, a group of 67 piglets with representative body weight and size were selected. Thirty-six animals, 22 IBxIB (9 females and 13 males) and 14 IBxLW (7 females and 7 males), were euthanized by stunning and exsanguination in compliance with RD 53/2013 and sampled at 60 days old (the end of the early juvenile period). Different samples were obtained and analyzed [23] and feces were obtained from the rectum. The remaining thirty-one animals, 18 IBxIB (9 females and 9 males) and 13 IBxLW piglets (6 females and 7 males), were moved to INIA-CSIC facilities and maintained in collective pens. Even though localization and diet changed (S2 Table), animals from both genotypes were always allocated in the same environmental conditions to avoid confounding factors. INIA-CSIC pig facilities consist of free-ranging pens with access to shadow areas in which genotypes and litters were mixed. At 210 days-old (end of the late juvenile period), the 31 pigs were euthanized by stunning and exsanguination in compliance with RD 53/2013 and sampled to obtain feces samples from the rectum, as well as other tissues [23]. The rectal content extracted at 60 and 210 days-old was immediately snap-frozen in liquid nitrogen and stored at– 80°C until the analyses were performed.

### Microbial DNA extraction and 16S rRNA gene sequencing

For each one of those 67 samples, DNA was extracted from 0.2 g rectal content using QIAamp PowerFecal® kit (QIAGEN, Hilden, Germany) according to manufacturer’s standard protocol. Illumina MiSeq® paired-end sequencing protocol (Illumina, San Diego, CA, USA) was performed by an external sequencing service (FISABIO bioinformatics, Valencia, Spain) targeting 16S rRNA gene V3-V4 amplicon. The employed primers were S-D-Bact-0341-b-S-17 and S-D-Bact-0785-a-A-21 [26] which produce amplicons of 464 bp. Raw microbial sequence data have been uploaded to the ENA repository and are available at https://www.ebi.ac.uk/ena/browser/view/PRJEB48905.

### Taxonomy classification and diversity studies

Bioinformatic analyses were performed using Quantitative Insights Into Microbial Ecology 2 (QIIME2) version 2019.10.0. [27] following author’s recommendations. In brief, raw fastq archives were imported to QIIME2 and then paired-end reads were joined, obtaining between 73979 and 207347 paired-reads per sample, which were processed into Amplicon Sequence Variants (ASVs) using DADA2 algorithm [28]. One sample corresponding to a 60 days-old IBxLW male piglet was excluded because of a very low reads number (n = 55]. Thus, the study was done with 66 animals. Taxonomy was studied using SILVA database 138. R version 4.2.3. [29] was utilized to further analyze the data.

The alpha and beta-diversities studies in the 66 samples were performed with the vegan R package version 2.6–2 after their rarefaction [30]. Plots to visualize diversities results were done with phyloseq 1.40.0 and ggplot2 3.4.1 [31]. Alpha-diversity calculates diversity within sample, attending to its richness (number of taxonomic groups, calculated by the Observed richness) and evenness (distribution of abundances of the groups, measured by the Shannon index) [32]. Both metrics were calculated using the function *estimate_richness* from phyloseq. Statistical significance was assessed with a two-way ANOVA that included genotype, age and sex and the genotype by age interaction.

Beta diversity indexes summarize which samples differs from one another by considering presence-absence sequences or sequence abundance [33]. To study beta diversity, Bray-Curtis dissimilarity index was calculated with phyloseq::distance function and were used, together with a Principal Coordinate Analysis (PCoA) plot. A PERMANOVA analysis using the adonis2 function within the vegan package was performed to ascertain significance, including genotype corrected by age and sex, as well as the genotype by age interaction.

Due to the strong age effect found, we subsampled the animals to analyze separately samples at 60 days-old (n = 35) and at 210 days-old (n = 31) using similar models as those described without the age effect. In all analyses, significance was considered when *p* < 0.05, whereas a tendency was observed when 0.05 < *p* < 0.1.

### Differential abundance analysis

Differential abundance analysis was performed after filtering samples with less than 30000 reads by using linear models within the linDA method [34] at ASV and genus level. The model included the genotype, sex and age effects. Microbiome of animals at 60 and 210 days-old were analyzed separately to better ascertain the effect of the genotype at each time-point. Significance was considered with a *p*-adjusted < 0.1.

### Short-chain fatty acid analysis from the rectal samples

Short-chain fatty acids (SCFA) from fecal samples were extracted in distilled water. Afterwards, the chromatographic analysis was performed using the method proposed by Zhao *et al*. [35]. In brief, an Agilent 6850N GC system equipped with a flame ionization detector (FID) and a N10149 automatic liquid sampler (Agilent, USA) and a DB-FFAP [30m x 0.25mm x 0.25μm) column (Agilent, USA) was used. Nitrogen was the carrier gas, with a constant pressure of 15 psi. Initial oven temperature was 100°C, until reaching 200°C, when this temperature was maintained for 5 minutes. Detector temperature was also 200°C, and hydrogen, air and nitrogen airflows were 40, 300 and 30 ml/min, respectively. We injected 1μl of sample, with a 5:1 split. Data analysis was done using Agilent ChemStation software (Agilent;USA). For statistical analysis, R version 4.2.3. was used to calculate two-way ANOVA considering genotype, sex, age and the genotype*age interaction to infer differences between IBxIB and IBxLW pigs, males and females and 60 and 210 days-old animals. Statistical significance was considered when *p* < 0.05, whereas a tendency was assumed when 0.05 < *p* < 0.1. SCFA, FA and body composition and abundance correlation was studied using a Pearson correlation corrected by a Bonferroni test in the mixOmics package 6.20.0. [36] in R, separating the samples by genotype and by age for a better understanding of the result.

## Results and discussion

In the present study, we aimed to determine possible differences in the gut microbiome related to the pig genotype by comparing pure Iberian (IBxIB) and Large White x Iberian crossbred (IBxLW) pigs gestated in the same Iberian mothers. In our design, and conversely to other studies comparing the gut microbiota between different swine breeds [37, 38], prenatal and early postnatal environments were the same and thus were presumably not affecting the possible differences in microbiota composition between genotypes. The maternal microbiome is crucial in the primocolonization of the offspring [39]. Therefore, the control of the maternal environment allows us to better understand direct genotype implications on microbiota at early (60 days-old) and late (210 days-old) juvenile growing phases.

As previously reported, these animals showed different phenotypic characteristics at both ages [23]. In brief, IBxIB piglets were significantly lighter and smaller than their IBxLW counterparts during all development. However, IBxIB animals had higher subcutaneous and intramuscular fat, while IBxLW pigs had higher muscle content, and heavier organs. The differences in adiposity also reflected the metabolic status (IBxIB animals showed dislipidemia) and fatty acid composition of the tissues (IBxIB animals had higher SFA content while IBxLW pigs showed higher PUFA content).

### Microbiota composition and diversity

**Fig 1:**
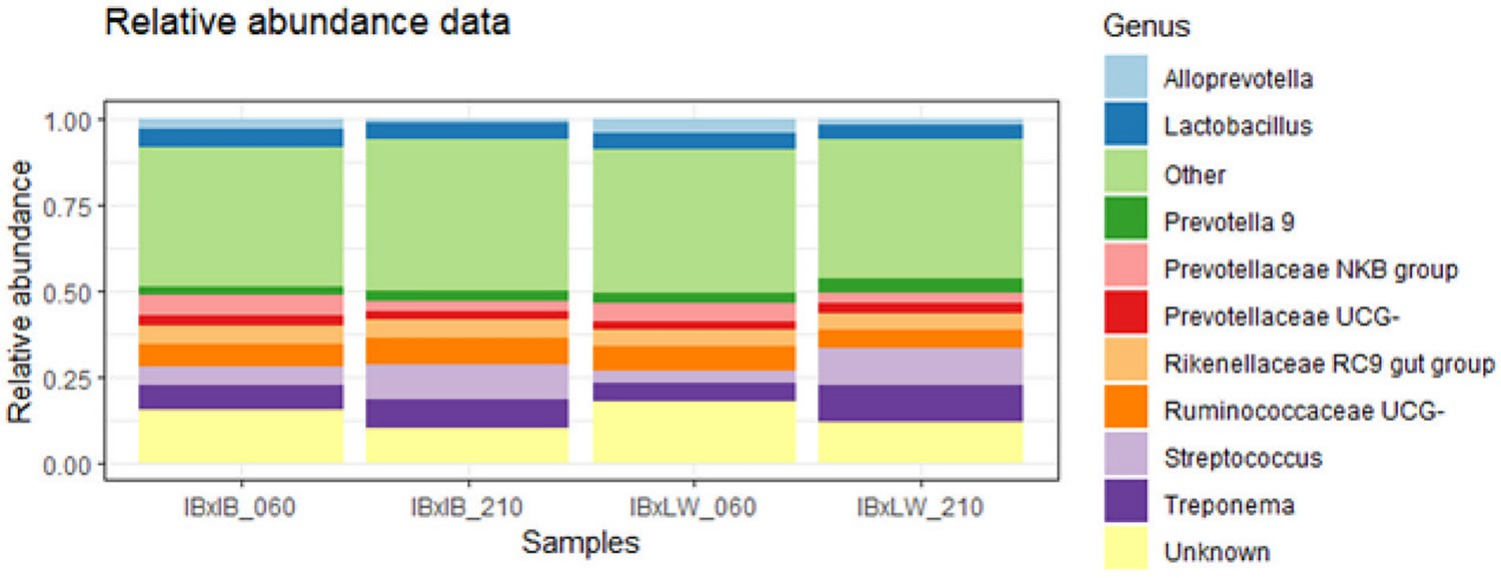
Main genera prevalence and abundance separated by genotype and age. IBxIB = pure Iberian pigs; IBxLW = Iberian*Large crossbreds; 060 = 60 days of age; 210 = 210 days of age.

The ASVs obtained during the analysis were aggregated into 20 phyla and 264 genera (Fig 1 and S3 and S4 Tables). *Firmicutes* and *Bacteroidetes* were the most prevalent phyla in both genotypes and time-points, in accordance with previous studies in pigs [17, 18]. At the genus level, at 60 days-old, *Treponema* and *Ruminococcaceae UCG-* were the most abundant genera in IBxIB pigs, while the most abundant genera in IBxLW pigs were *Ruminococcaceae UCG-* and *Prevotellaceae NKB group*. At 210 days-old, the most abundant genera in both genotypes were *Streptoccocus* and *Treponema*. The high abundance of *Treponema* is in accordance with other studies in Iberian pig [17]. Even if animals in the study conducted by Crespo-Piazuelo *et al*. [17] were older, *Treponema* was the second most abundant genus in rectal samples. On the other hand, *Prevotella* is one of the most abundant genera in pigs [40] and precisely *Prevotella* enterotype has been related to traits such as weight gain especially after weaning [41]. Thus, this difference in the *Prevotella* and *Treponema* proportions between genotypes could be related to their difference in average daily weight gain [23], since *Prevotella* has been related to better weight gain [42]. The high presence of *Streptococcus* in porcine samples is uncommon. However, we should consider firstly that SILVA database subdivides genera (thus, appearing under different designations in our results). Also it is important to note that differences between studies are driven by multiple variables, from the environment and physiological characteristics of the animals to the reagents used in the posterior extraction of DNA or data analysis [43].

Alpha diversity refers to the diversity within a sample. Alpha diversity measures (Observed richness and Shannon index) were calculated due to their correlation with host resilience [44], adiposity [45] or feed efficiency [46]. Thus, the Iberian pig, being a rustic breed is more resilient than commercial pigs like the Large White [47]. Furthermore, as stated before, our IBxIB animals were fattier than their crossbred littermates [23], and fatty pigs have lower feed efficiency than lean ones [48].

**Fig 2:**
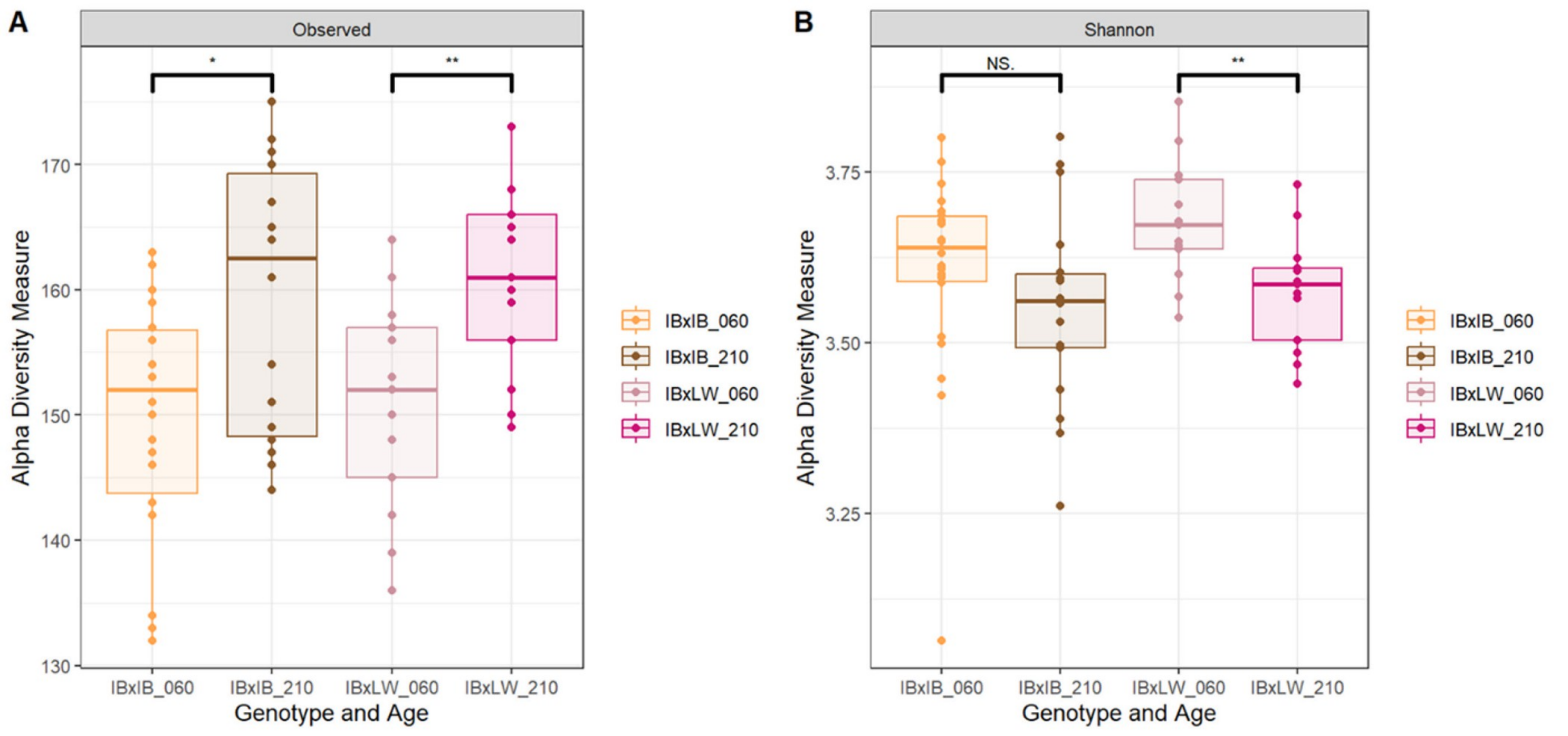
Alpha diversity indexes (Observed richness Shannon index) by genotype and age at the genus level. IBxIB pure Iberian pigs; IBxLW = Iberian*Large White crossbreds; 060 = 60 days-old, 210 = 210 days-old. *p < 0.05; **p < 0.01.

Therefore, it was hypothesized that differences in these indexes could help to explain some of the phenotypic differences between the IBxIB and IBxLW pigs. However, contrary to previous research, genotype did not affect alpha diversity in our study (Fig 2). A plausible explanation would be the common maternal environment shared by the animals in the present research due to its importance in the microbiome composition throughout the litter lifespan [49]. Thus, in other studies, the breed effect was tested in animals with mothers of different breeds [18, 37, 38], which may explain the differences between our study and others.

Age had a significant effect on Observed richness both at the ASV and Genus level (assessed with a two-way ANOVA; *p* < 0.001). These results coincide with previous research in which Observed richness increased with age in pigs [50, 51]. In the Shannon index, only IBxLW pigs showed significant differences between ages (*p* < 0.01). The lack of significance in the IBxIB pigs could be due the higher variability found in this group. Interestingly, studies show an increase of the Shannon index with time, even in pigs with similar ages as those used in the present research [52], while our results showed lower values at 210 days. This outcome could be related to the change of farms performed before the sampling of piglets at 60 days, since an environmental change can affect the alpha diversity [53]. Sex did not affect alpha diversity.

**Fig 3:**
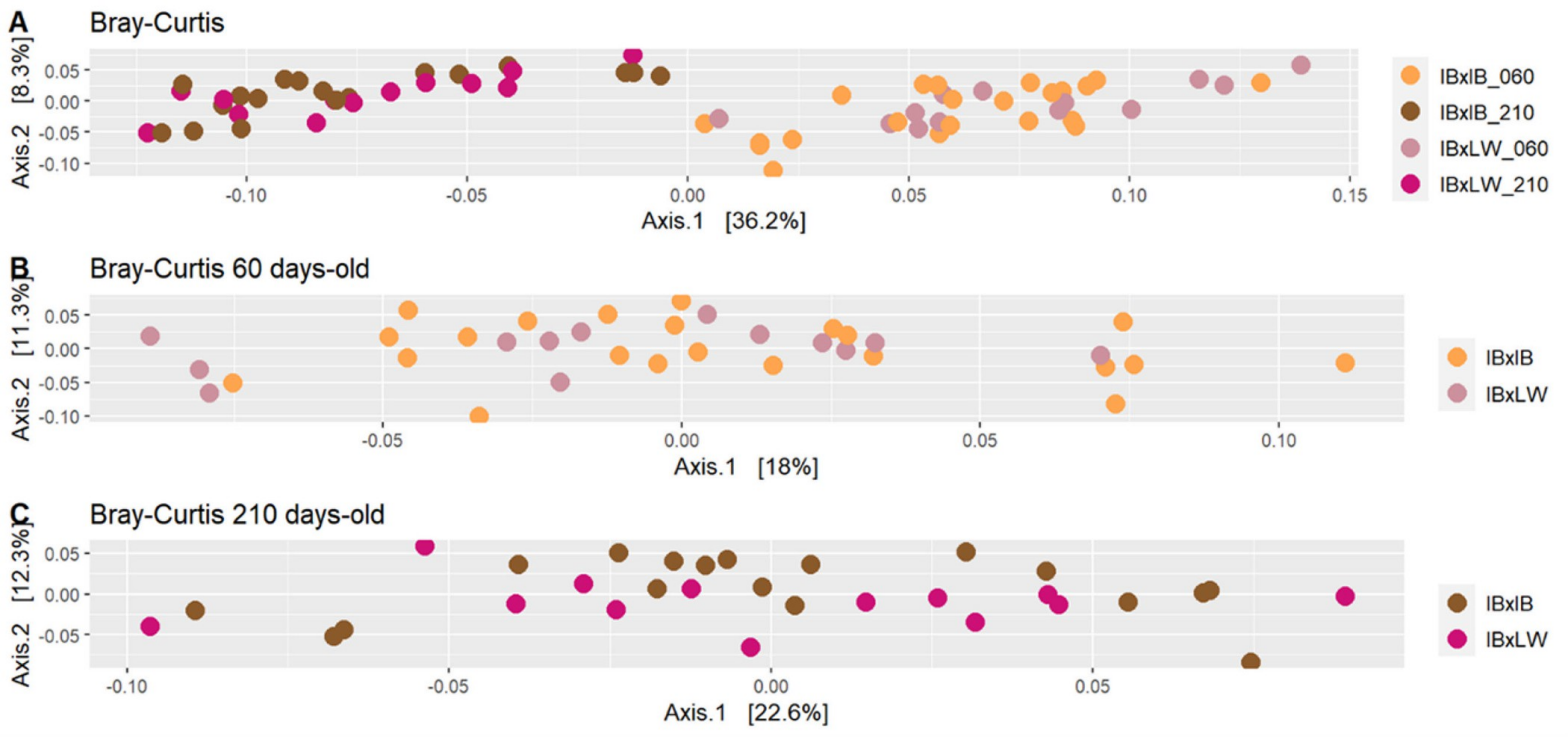
Principal Coordinate Analysis (PCoA) plot based on Bray-Curtis beta diversity indexes by genotype and age (panel A), by genotype at 60 days-old (panel B) and by genotype at 210 days-old (panel C). IBxIB pure Iberian pig animals; IBxLW = Iberian*Large White crossbreds; 060 = 60 days-old, 210 = 210 days-old.

Beta-diversity (diversity between samples) was studied using Bray Curtis index (Fig 3). When using the total number of pigs, samples were clustered according to animals’ age, which had a significant effect (*p* < 0.001). This is in accordance with age having important implications on the microbiome profile during the pig’s lifetime, in addition to the changes in environment and diet previously mentioned [54]. There were no differences between sexes. When the genotype effect was analyzed using all samples, there were differences between IBxIB and IBxLW pigs (*p* = 0.08). When each time-point was studied separately, there were significant differences between genotypes both at 60 and 210 days-old (*p* < 0.05]. Although results were similar using samples at ASV and Genera levels, significance was only achieved at the ASV level. Differences between genotypes in the beta diversity indexes, implicating differences between microbiomes of both genotypes, could help to explain the different metabolism observed in both genotypes, particularly in adipogenesis and growth rates, especially in older animals [23], since microbiota composition has been related to adiposity and obesity [55, 56] and feed efficiency [57].

### Differential abundance of the rectal microbiome composition

There were three ASVs with significantly different abundances between genotypes. Thus, an ASV from the genus *Oscillibacter*, a genus correlated with lower feed efficiency, was overabundant in IBxIB pigs [19]. This is in agreement with the lower average daily weight gain observed in IBxIB in comparison to IBxLW pigs [23], and furthermore, other works have proved Iberian pigs have lower feed efficiency than highly-selected breeds like the Large White [58]. The two others differentially abundant ASVs corresponded to *Ruminococcaceae UCG-* and *NKA* genera, respectively, being overabundant in IBxLW genotype. The *Ruminococcacea* genera includes various polysaccharide fermenters and also participates in methanogenesis by producing H2 and formate, which serve as substrates for archaeal communities [59]. Therefore, they might be relevant for traits related to metabolism [18]. Age, on the other hand, had a strong effect on microbiota composition, as expected, with more than 1800 ASVs being differentially abundant between pigs at 60 and 210 days-old (S5 Table). This result is in accordance with previous research and expected, since age is one of the most important factors modulating microbiota composition [50].

Analyses were also conducted agglomerating the ASVs into genus. In this regard, 6 genera showed different abundance between genotypes (*p*-adjusted < 0.1; Table 1). All of them were overabundant in IBxLW pigs except for *Candidatus Soleaferrea*, which was overabundant in IBxIB animals. This genus has not yet been fully studied in pigs, so further research is needed to understand its possible role in animal metabolism and in the differences found between genotypes. However, it has been previously associated with lower body weight in chickens [60], which would be in accordance with phenotypic results in which IBxIB pigs were lighter than their IBxLW counterparts. Among the genera overabundant in IBxLW pigs, *Anaerovibrio* has been found negatively associated with fatness traits in pigs [19]. Therefore, this finding might match the lower adiposity found in IBxLW pigs compared to IBxIB animals. Again, there were no differences between sexes, while the age effect at the genera level was stronger than the genotype effect, with 130 genera being differentially abundant between pigs at 60 and 210 days-old (S6 Table).

**Table 1:** Differences in genera abundance in fecal samples between pure Iberian (IBxIB) and Iberian*Large White crossbreds (IBxLW).

Genus	logFC^a^	Overabundance	pvalue	padj
*GCA-9*	0.822	IB*LW	0.001	0.068
*Anaerovibrio*	1.040	IB*LW	0.000	0.068
*uncultured rumen bacterium*	1.794	IB*LW	0.001	0.068
*gut metagenome*	1.762	IB*LW	0.001	0.068
*uncultured rumen bacterium C8d-*	1.271	IB*LW	0.002	0.083
*Candidatus Soleaferrea*	-0.517	IB*IB	0.003	0.098

^a^FC = Fold Change

Due to this strong age effect, analyses with LinDA were also performed separately for 60 and 210 days-old animals. At 60 days-old, no differences were found between genotypes at the ASV level, while at the genus level only *Terrisporobacter* was overabundant in IBxIB pigs (p-adjusted < 0.1]. However, at 210 days-old, there were differences in the abundance of 12 ASVs and 7 genera (Tables 2 and 3). The IBxIB group had higher levels of ASVs from *Oscillibacter*, *Christensenellaceae R-7 group* and *Ruminococcaceae UCG-9* while ASVs from the *Ruminococcaceae NKA group* and *Treponema* were overabundant in IBxLW pigs (Table 2). As stated before, *Oscillibacter* is related to lower feed efficiency, and can correspond to the lower average daily weight gain found in IBxIB animals, especially at older ages. However, *Christensenellaceae* family has been repeatedly associated with regular Body Mass Index and leanness in humans and lower lipids in serum, as reviewed in [61]. Furthermore, it has been observed to be increased in pigs with high conversion ratios [62]. IBxIB pigs had higher adiposity levels, especially at the oldest age, and higher levels of cholesterol (both HDL-c and LDL-c) in plasma at this age [23]. Therefore, further research is needed to fully understand this outcome. The finding of higher *Treponema* abundance in IBxLW animals could be related to their lower MUFA and oleic acid content, as observed by López-García *et al*. [18].

**Table 2:** Differentially abundant ASVs between pure Iberian (IBxIB) and Iberian*Large White crossbred pigs (IB*LW) at 210 days-old.

Genus	ASV	log2FC	Overabundance	pvalue	padj
*Oscillibacter*	337	-2.992	IBxIB	0.000	0.045
*Christensenellaceae R-7 group*	1068	-2.661	IBxIB	0.000	0.045
*Christensenellaceae R-7 group*	278	-2.452	IBxIB	0.000	0.067
*Z*	38	-2.152	IBxIB	0.000	0.073
*NA*	1327	-1.992	IBxIB	0.000	0.076
*Ruminococcaceae UCG-*	2220	-1.801	IBxIB	0.000	0.073
*uncultured*	2603	-1.622	IBxIB	0.000	0.099
*NA*	1703	1.273	IBxLW	0.000	0.073
*Treponema*	133	1.277	IBxLW	0.000	0.064
*Ruminococcaceae NKA group*	179	2.089	IBxLW	0.000	0.034
*uncultured rumen bacterium C8d-*	2500	2.168	IBxLW	0.000	0.073
*NA*	2750	2.295	IBxLW	0.000	0.064

**Table 3:** Differentially abundant genera between pure Iberian (IBxIB) and Iberian*Large White crossbred pigs (IB*LW) at 210 days-old.

Genus	log2FC	Overabundance	pvalue	padj
*Family XIII A group*	-0.905	IBxIB	0.000	0.022
*Ruminococcaceae UCG-*	-0.779	IBxIB	0.000	0.022
*Candidatus Soleaferrea*	-0.919	IBxIB	0.001	0.024
*Ruminococcaceae UCG-9*	-0.986	IBxIB	0.000	0.024
*uncultured rumen bacterium C8d-*	2.150	IBxLW	0.000	0.024
*metagenome*	-1.346	IBxIB	0.002	0.068
*Ruminococcaceae NKA group*	-0.674	IBxIB	0.003	0.086

When the analysis at 210 days-old was performed aggregating results at the genus level (Table 3), the most important result was the overabundance of *Ruminococcaceae UCG-* groups in IBxIB animals, with phenotypic implications explained before.

Even though some differences were found when comparing the microbiota composition, these were lower in number and less significant than in previous studies comparing the microbiome composition of different pig breeds [18, 19]. Firstly, it must be considered that other approaches were used, since the study by Lopez-García *et al*. used OTUS and Deseq2 or ALDEX2. Bergamaschi *et al*. utilized the ASV approach, but afterwards, data was analyzed using edgeR. Furthermore, in both articles, each breed was gestated in its corresponding mother, so the maternal effect, with great importance in microbiota primocolonization [39] and with long-lasting effects on the animal [21] could not be left aside. However, further research should be carried out on a larger number of animals to fully determine the impact of breed on the microbiota composition without prenatal effects and its consequences on meat characteristics and productivity.

### Short-chain fatty acids (SCFA) in feces

Given the differences in microbiota and fatty acid composition in different tissues [23], we explored whether these animals displayed differences in the short-chain fatty acid (SCFA) composition of the faeces. SCFAs have important implications on digestibility [63], adiposity and obesity [64] as well as in body-weight [65].

In the present study, we did not find differences between genotypes nor sexes; but there was a strong effect of age on SCFAs. Thus, acetic acid was higher in the 210 -days-old compared to 60 days-old animals, and propionic acid was higher in the younger animals (*p* < 0.05 for both). A limitation of the present study was that SCFA concentration in the bloodstream was not analysed since their easy and rapid absorption [66] may explain the scarce genotype differences at the faeces level.

### Microbiome and phenome correlations

To further investigate the possible relation of the microbiome with the phenotypic differences found between genotypes, correlations with body weight, tissue fatty acid composition, adiposity and SCFA of faeces were performed in each genotype and age separately (Tables 4–7). Previous research has demonstrated the influence of a stable microbiota in muscle quantity and quality [67–69] as well as its implications in liver metabolism and fatty acid composition [70]. As stated before, there were multiple differences in fatty acid composition between genotypes [23], possibly due to the higher adipose tissue synthesis the Iberian pig [71]. Furthermore, SCFAs have been related to metabolic syndrome and other metabolic diseases. The Iberian pig has a natural state similar to this syndrome,so it was also of our interest to further study the possible relation between the genera found in fecal samples and the SCFA of those samples.

**Table 4:** Correlations between different genera obtained from fecal samples and phenotypical traits in pure Iberian (IBxIB) pigs at 60 days-old.

Correlation		r	p	p-adjusted
Fatty acids of the neutral fraction of the liver				
PUFA^a^	*Mollicutes RF9-*	0.840	0.000	0.007
	*[Eubacterium] eligens group*	0.788	0.000	0.019
n3 PUFA	*Mollicutes RF9-Hepatic*	0.807	0.000	0.013
	*[Eubacterium] eligens group*	0.788	0.000	0.019
n6 PUFA	*Mollicutes RF9-Hepatic N6 A*	0.827	0.000	0.007
	*[Eubacterium] eligens group*	0.771	0.000	0.031

^a^PUFA: Polyunsaturated Fatty Acid

**Table 5:** Correlations between different genera obtained from fecal samples and phenotypical traits in Iberian and Large White (IBxLW) crossbred pigs at 60 days-old.

Correlation		r	p	p-adjusted
Short chain Fatty Acids in Fecal samples				
Acetic acid	*Megasphaera*	0.900	0.000	0.023
	*Cellulosilyticum*	0.865	0.000	0.023
	*Intestinibacter*	0.865	0.000	0.023
	*Enitrobacterium*	0.865	0.000	0.023
	*Acidaminococcus*	0.845	0.000	0.038
	*Pyramidobacter*	0.807	0.001	0.098
Fatty acids of the neutral fraction of the Longissimus dorsi muscle				
n3 PUFA^a^	Anaerovibrio	0.888	0.000	0.037
Fatty acids of the polar fraction of the Longissimus dorsi muscle				
SFA^b^	[Eubacterium] hallii group	-0.888	0.000	0.037
	Corynebacterium	-0.867	0.000	0.062
	Puniceicoccaceae	-0.867	0.000	0.062
	Cerasicoccus	-0.867	0.000	0.062
MUFA^c^	Corynebacterium	0.919	0.000	0.019
	gut metagenome	0.910	0.000	0.019
	Mucispirillum	0.919	0.000	0.019
	Cerasicoccus	0.919	0.000	0.019
	uncultured rumen bacterium	0.919	0.000	0.019
	Oxalobacter	0.863	0.000	0.067
PUFA^a^	gut metagenome	-0.912	0.000	0.019
	Cerasicoccus	-0.909	0.000	0.019
	Corynebacterium	-0.909	0.000	0.019
	Mucispirillum	-0.909	0.000	0.019
	Mucispirillum	-0.909	0.000	0.019
	uncultured rumen bacterium	-0.909	0.000	0.019
n6 PUFA^a^	gut metagenome	-0.920	0.000	0.019
	Corynebacterium	-0.917	0.000	0.019
	Mucispirillum	-0.917	0.000	0.019
	Cerasicoccus	-0.917	0.000	0.019
	uncultured rumen bacterium	-0.917	0.000	0.019
Fatty acids of the neutral fraction of the liver				
SFA^b^	Faecalibacterium	0.853	0.000	0.092
PUFA^a^	uncultured bacterium	0.896	0.000	0.031
	Bilophila	0.896	0.000	0.031
n3 PUFA^a^	gut metagenome	0.893	0.000	0.034
n6 PUFA^a^	Bilophila	0.849	0.000	0.097
	Oxalobacter	-0.878	0.000	0.051
	Prevotellaceae NKB group	-0.869	0.000	0.062
Fatty acids of the polar fraction of the liver				
MUFA^c^	Candidatus Melainabacteria bacterium MEL.A	-0.892	0.000	0.035
	Enterococcus	-0.881	0.000	0.046

^a^PUFA = Polyunsaturated Fatty Acids

^b^SFA: Saturated Fatty Acids

^c^MUFA: Monounsaturated Fatty Acids

**Table 6:** Correlations between different genera obtained from faecal samples and phenotypical traits in pure Iberian (IBxIB) pigs at 210 days-old.

Correlation		r	p	p-adjusted
Fatty acid of the neutral fraction of the Longissimus dorsi muscle				
PUFA^a^	*Atopobiaceae*	0.889	0.000	0.005
	*Acinetobacter*	0.847	0.000	0.019
n3 PUFA^a^	*Elusimicrobium*	0.884	0.000	0.005
n6 PUFA^a^	*Atopobiaceae*	0.872	0.000	0.007
	*Acinetobacter*	0.824	0.000	0.043

^a^PUFA = Polyunsaturated Fatty Acids

**Table 7:** Correlations between different genera obtained from fecal samples and phenotypical traits in Iberian and Large White (IBxLW) crossbred pigs at 210 days-old.

Correlation		r	p	p-adjusted
Body weight	*[Eubacterium] xylanophilum group*	-0.855	0.000	0.074
Subcutaneous fat (Total; mm)	*Moryella*	-0.862	0.000	0.074
Subcutaneous Fat (Inner layer; mm)	*Muribaculaceae*	-0.886	0.000	0.064
Longissimus dorsi (mm)	*Pirellulaceae*	-0.840	0.000	0.092

In Table 4, the significant correlations in IBxIB animals at 60 days old are presented. All the positive correlations found in this group are related to the PUFA (both n3 and n6] in the liver. *Mollicutes* and *[Eubacterium] eligens group* have been previously related to liver disease and cancer [72–74]. Therefore, they could be related to the liver metabolism in the Iberian pig and its differences with other pig breeds [75].

In IBxLW pigs at 60 days old (Table 5), different genera were positively related to the acetic acid concentration in feces. In fact, acetate has been identified as beneficial in host metabolism by binding with different G-protein coupled receptors [76], by being transformed in acetyl-CoA and incorporated in the tricarboxylic acid cycle [77] or affecting the 5’ AMP-activated protein kinase to increase oxidative capacity [78]. IBxLW pigs showed higher average daily weight gains and muscle accretion than their IBxIB counterparts [23], so these genera and their relationship with the acetic acid production could be a factor in the growth difference. However, no significant differences were found in the concentration of acetic acid in the faecal samples. It must be considered that SCFAs are rapidly absorbed in the colon, being one of the limitations of the present study the lack of data regarding SCFA in plasma.

On the other hand, many genera have been related to fatty acid composition of both muscle and liver. Commercial farm pigs have been selected to reduced fat accumulation and lipogenesis, so they resemble better the fatty acid composition of the feed than rustic breeds [71]. In pigs, an interrelation between gut microbiota and fatty acid composition of the tissues have been found [79], so these correlation could partially explain the differences in fatty acid composition between IBxIB and IBxLW pigs.

Interestingly, correlations found in IBxIB animals at 210 days-old (Table 6) are also related to PUFA content, although in the muscle instead of the liver. This could indicate the importance of microbiota in modulating PUFA in different tissues, since, as stated before, the microbiota is related to fatty acid composition of the animals [79]. *Acinetobacter* is a bacteria which shows a great activity of desaturases and, therefore, unsaturated fatty acid production in order to survive [80]. *Atopobiaceae* has been related to the ingestion of high fat diets and, therefore, higher adiposity, in accordance with the higher adiposity of the Iberian pig. On the other hand, *Elusimicrobia* has been linked to more maigre animals [81], so its correlation with n3 PUFA in Iberian pigs must be further studied.

In Table 7, correlations between genera and adiposity and growth are shown. Since IBxLW pigs, especially at 210 days old, were heavier and meatier than IBxIB animals, the correlation with *[Eubacterium] xylanophilum* group was negative. *[Eubacterium] xylanophilum* has been negatively associated with body weight before [82]. On the other hand, Pirellulaceae has been positively associated with weight gain [83], although is not explained if it is in fat or muscle content. *Moryella* and *Muribaculaceae* were negatively associated with fat content, in accordance with the leanness of the IBxLW pigs.

This study is, to the best of our knowledge, the first attempt to identify microbiome composition differences due to genotype excluding pre and postnatal environmental factors. Results show differences driven by only genotype between a rustic, fatty pig (the Iberian pig) and a more selected and maigre breed (Large White) gestated in the same mothers. These differences in the microbiome composition could be responsible for differences in the phenotype, such as growth patterns or adiposity. In fact, significant correlations have been found in both genotypes and at both ages [60 and 210 days-old). These correlations mainly connected the microbiome with fatty acid composition of different tissues, especially PUFA content. Previous research also shows a relation between PUFA and microbiota, in which PUFA can alter microbiota composition while microbiota can affect metabolism and absorption of PUFA [84, 85], although further investigation is necessary to fully understand this link between PUFA composition and microbiota. Thus, the selection of certain microbiota within animals, which may affect the adiposity, the growth pattern or even the meat quality could be a useful tool in the future [86].

Major limitations of the present study must be considered. The most important is the sample size used, which may be limiting for the variables studied. Therefore, a greater number of animals per group are required to detect a higher number of abundance differences, especially considering the individual variation in microbiome composition [87]. Furthermore, this research shows differences only at two time-points, so studies at different ages would be also interesting to see how genotypes modulate microbiome throughout life and which implications could have the selection of certain microbiota in different breeds.

## Conclusions

The present study supports previous results of host genetic effects on gut microbiota composition in pigs. In our study, ASV from the *Oscilibacter* genus, as well as genera such as *Ruminococcaceae UCG*-, *Anaerovibrio* or *Candidatus Soleaferra* showed different abundances between genotypes. The fact that animals used were gestated in the same maternal environment provides additional relevance to the difference observed among breeds since implications of different prenatal and postnatal environments were eluded. These results help to understand underlying differences in microbiota that could explain differences in phenotype, especially related to growth rates and adiposity.

## Supporting information

S1 Table Calculated analysis (g/kg, dry-matter basis) and ingredients (%) of the gestating sows diet. (XLSX)

S2 Table Calculated analysis (g/kg, dry-matter basis) and fatty acid composition of the piglets diets. (XLSX)

S3 Table List of phyla found in the microbiome of IBxIB and IBxLW animals. (XLSX)

S4 Table List of genera found in the microbiome of IBxIB and IBxLW animals. (XLSX)

S5 Table Differentially abundant ASV between pure Iberian and Iberian*Large White crossbreds at 60 and 210 days-old. (XLSX)

S6 Table Differentially abundant genera between pure Iberian and Iberian*Large White crossbreds at 60 and 210 days-old. (XLSX)
